# Improving Breakdown Voltage and Threshold Voltage Stability by Clamping Channel Potential for Short-Channel Power p-GaN HEMTs

**DOI:** 10.3390/mi13020176

**Published:** 2022-01-25

**Authors:** Hongyue Wang, Yijun Shi, Yajie Xin, Chang Liu, Guoguang Lu, Yun Huang

**Affiliations:** 1 Science and Technology on Reliability Physics and Application of Electronic Component Laboratory, China Electronic Product Reliability and Environmental Testing Research Institute, Guangzhou 510610, China; syijun0119@163.com (Y.S.); xd_liuchang@163.com (C.L.); luguoguang@ceprei.com (G.L.); hyun@ceprei.com (Y.H.); 2 State Key Laboratory of Electronic Thin Films and Integrated Devices, University of Electronic Science and Technology of China (UESTC), Chengdu 610054, China

**Keywords:** breakdown voltage, partially recessed, p-GaN HEMT, short-channel

## Abstract

This paper proposes a novel p-GaN HEMT (P-HEMT) by clamping channel potential to improve breakdown voltage (BV) and threshold voltage (*V*_TH_) stability. The clamping channel potential for P-HEMT is achieved by a partially-recessed p-GaN layer (PR p-GaN layer). At high drain bias, the two-dimensional electron gas (2DEG) channel under the PR p-GaN layer is depleted to withstand the drain bias. Therefore, the channel potential at the drain-side of the p-GaN layer is clamped to improve BV and *V*_TH_ stability. Compared with the conventional p-GaN HEMT (C-HEMT), simulation results show that the BV is improved by 120%, and the *V*_TH_ stability induced by high drain bias is increased by 490% for the same on-resistance. In addition, the influence of the PR p-GaN layers’ length, thickness, doping density on BV and *V*_TH_ stability is analyzed. The proposed device can be a good reference to improve breakdown voltage and threshold voltage stability for short-channel power p-GaN HEMTs.

## 1. Introduction

GaN-based devices are promising for next-generation high-efficiency, high-frequency, high-temperature, and high-power applications due to their superior material properties [1,2,3,4,5,6,7,8]. According to the application requirements, it is necessary to improve the electric performance of GaN devices [9].

For power applications, low on-resistance *R*_on_ and high breakdown voltage (BV) for GaN HEMTs are very desirable [7]. In order to realize low on-resistance, a short length scheme is always chosen as channel resistance under the gate is the main part of the total resistance for AlGaN/GaN HEMTs [10]. However, the short channel GaN HEMTs often suffer from the adverse drain-induced barrier lowering (DIBL) effect [11], namely, degradation of forward-blocking characteristics and negative threshold voltage (*V*_TH_) shift at high drain bias [12,13]. In order to suppress the DIBL effect-induced BV degradation, Pinchbeck et al. proposed a GaN HEMT with extended gate length to achieve reduced short channel effect and improved BV [14]. In addition, Lu et al. proposed a dual gate AlGaN HEMT to achieve high BV, low on-resistance, and high threshold voltage characteristics [15]. However, those methods are not suitable for short-channel p-GaN HEMTs to suppress the BV degradation and *V*_TH_ instability, which owns a p-GaN layer to achieve enhancement-mode function.

In this work, we proposed a novel p-GaN HEMT to improve the BV and *V*_TH_ stability, which features a partially-recessed p-GaN layer. At high drain bias, the two-dimensional electron gas (2DEG) channel under the partially-recessed p-GaN layer can withstand the high drain voltage to achieve higher BV and more stable *V*_TH_ for the short-channel p-GaN HEMTs. The paper is organized as follows: the device structure and operation mechanism of the proposed p-GaN HEMT are presented in Section 2; the simulation results and discussions are shown in Section 3; the conclusions are drawn in Section 4.

## 2. Device Structure and Mechanism

**Figure 1:**
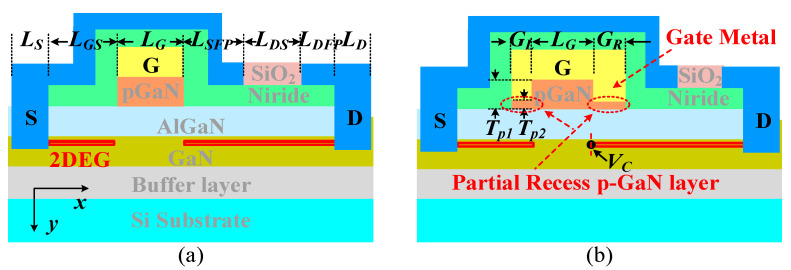
The schematic device structures of (**a**) conventional p-GaN HEMT (C-HEMT) and (**b**) proposed p-GaN HEMT (P-HEMT) with partially-recessed p-GaN layer (PR p-GaN layer).

**Figure 2:**
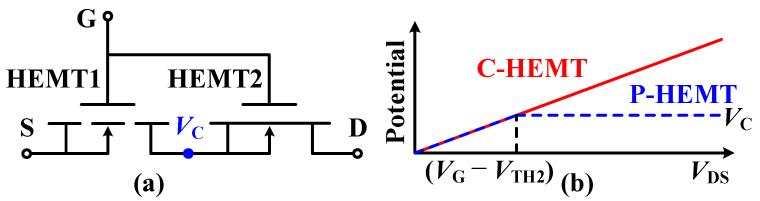
(**a**) The equivalent model of the P-HEMT with a high threshold voltage HEMT1 and a low threshold voltage HEMT2; (**b**) the potential *V*_C_ versus *V*_DS_.

The schematic structure of the proposed p-GaN HEMT (P-HEMT) is shown in Figure 1b. Compared with conventional p-GaN HEMT (C-HEMT), the P-HEMT features a partially recessed layer (PR p-GaN layer). To illustrate the mechanism of improving BV and *V*_TH_ stability for the P-HEMT, we employ one equivalent model with two series HEMTs, which are defined as high threshold voltage HEMT1 and low threshold voltage HEMT2, as shown in Figure 2a. As the threshold voltage of HEMT1 (*V*_TH1_) is larger than the threshold voltage of HEMT2 (*V*_TH2_), HEMT2 has been turned on when the gate to source voltage (*V*_GS_) is larger than *V*_TH1_. Therefore, the threshold voltage *V*_TH_ of P-HEMT is mainly determined by *V*_TH1_, namely, *V*_TH_ ≈ *V*_TH1_. The potential is defined as *V*_C_ at the connection node, which is also shown in Figure 1b. When 0 < *V*_C_ < *V*_GS_—*V*_TH2_ (i.e., *V*_GS_—*V*_C_ > *V*_TH2_), HEMT2 is at on-state. Therefore, *V*_C_ increases with *V*_DS_ at low drain bias. When *V*_C_ > *V*_GS_—*V*_TH2_ (i.e., *V*_GS_—*V*_C_ < *V*_TH2_), HEMT2 is in an off-state and the 2DEG channel under the PR p-GaN layer is depleted to withstand *V*_DS_ voltage. Therefore, *V*_C_ is clamped and does not increase with *V*_DS_ at high drain bias, as the blue dash line shown in Figure 2b. As a result, the barrier height for electrons injecting from source to drain will be hardly influenced by high drain bias, which makes *V*_TH_ more stable. In addition, the stable barrier height leads to decreased electrons flowing from source to drain compared with C-HEMT at high drain bias, which induces delayed occurrence of avalanche breakdown, namely, improves breakdown voltage.

## 3. Results and Discussions

**Figure 3:**
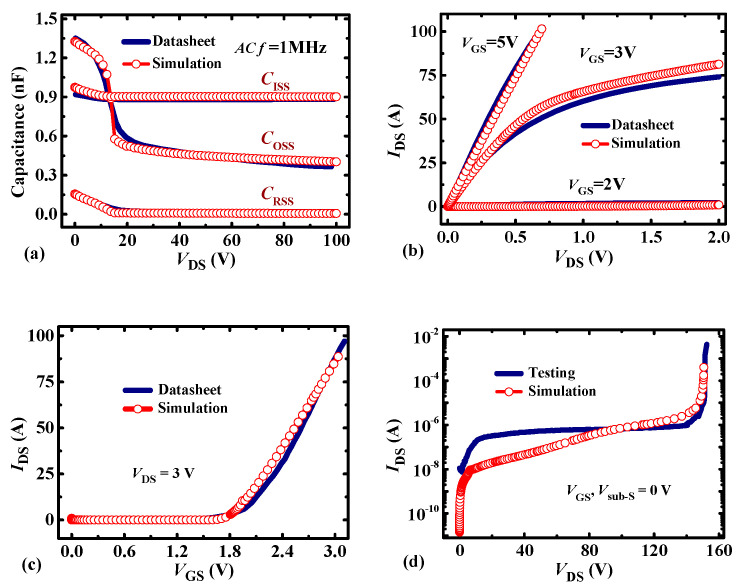
(**a**) Capacitance-Voltage characteristic; (**b**) output characteristic; (**c**) transfer characteristic; (**d**) forward-blocking characteristic. The forward-blocking characteristic is based on the testing data as there is no breakdown voltage result in the datasheet.

In this section, the current-voltage and capacitance-voltage characteristics of P-HEMT are investigated by Sentaurus TCAD simulation software [16], and the design considerations are also discussed. In the simulation, the optimized device parameters are as listed in Table 1 unless otherwise specified, which is also based on our previous calibrated work [17]. In particular, the structure parameters of C-HEMT are designed according to the dissected cross-sectional scanning electron microscope (SEM) images. The x- and y-coordinates and the epitaxial structures of the two devices are illustrated in Figure 1 [18]. For C-HEMT, an ionized acceptor concentration *N*_p-GaN_ = 3.5 × 10^17^ cm^−3^ is induced in the p-GaN layer with the *t*_p-GaN_ = 50 nm, which contributes to *V*_TH_ and on-state current calibrations for the C-HEMT. In addition, the deep acceptor traps and self-compensating donor traps [19] are also considered in the AlGaN buffer layer with an activation energy of *E*_V_ + 0.9 eV and *E*_C_—0.11 eV [20], and the trap density is 3 × 10^16^ cm^−3^ and 1.3 × 10^15^ cm^−3^ respectively [21]. Typically, the *G*_L_ of the PR layer on the source side is only set to 0.1 μm considering the deviation of the fabrication process, and it should be as small as possible to reduce the negative influence on input capacitance in practical application. The *G*_R_ of the PR layer on the drain side is an adjustable parameter as it makes obvious significance on the improvement of BV and *V*_TH_ stability. In this paper, the gate length *L*_G_ of C-HEMT is the same as the length of the thicker p-GaN layer of P-HEMT for achieving the same on-resistance, and the length of the partially-recessed p-GaN layer is not included in the nominal gate length *L*_G_. Table 1 shows the calibrated results of the 100 V enhancement-mode p-GaN HEMT [22], and it can be seen that the results are in good agreement with the datasheet as shown in Figure 3. Typically, the BV characteristic considering the avalanche model [23] coincides well with the testing result.

### 3.1. Static and Transient Characteristics

**Figure 4:**
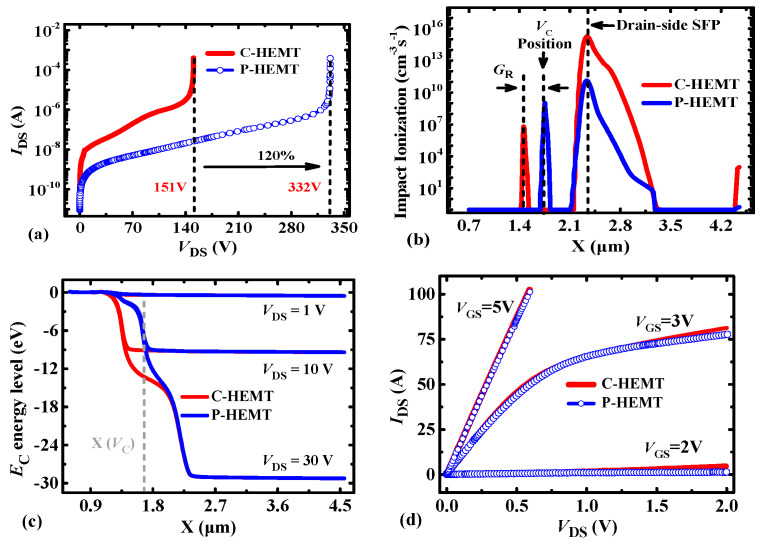
Comparison of the (**a**) forward-blocking characteristic, (**b**) impact ionization profile along the channel at *V*_DS_ = 150 V, (**c**) conduction energy level *E*_C_ profile along the channel, and (**d**) output characteristic between C-HEMT and P-HEMT.

Figure 4 shows the forward-blocking and output characteristics of the P-HEMT. As shown in Figure 4a, it can be seen that the BV (*I*_DS_off_ = 100 μA) for the P-HEMT is increased by 120% compared with the 100 V C-HEMT, which mainly results from the delayed occurrence of avalanche breakdown. As shown in Figure 4b, it can be observed that impact ionization at the drain-side source field plate is decreased at the same 150 V drain bias, which results from the decreased electrons flowing from source to drain. In addition, as shown in Figure 4c, the conduction energy *E*_C_ level at the drain-side of the p-GaN layer for P-HEMT is clamped, which results from the clamped *V*_C_ as stated in section II. As shown in Figure 4d, for typical gate operation voltage *V*_GS_ = 5 V, the output curves of C-HEMT and P-HEMT are coincident well, which indicates that the PR p-GaN layer makes a negligible impact on the on-state resistance. For *V*_GS_ = 2 V, the *I*_DS_ for C-HEMT is slightly higher than P-HEMT, which results from the partial depletion of the 2DEG channel under the PR p-GaN layer.

**Figure 5:**
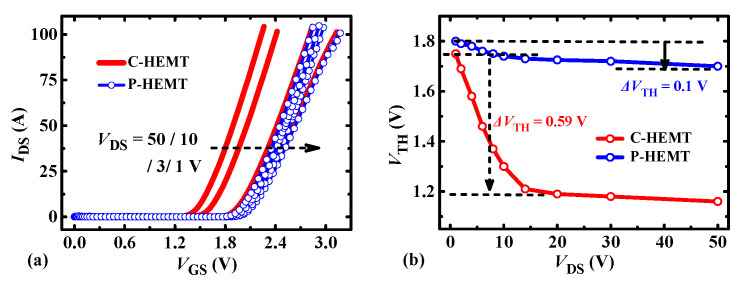
Comparison of (**a**) the transfer, (**b**) the threshold voltage *V*_TH_ (at *I*_DS_ = 10 mA) depending on *V*_DS_ between the C-HEMT and P-HEMT. For a fair comparison, the *V*_TH_ is defined when *I*_DS_ =10 mA.

Figure 5 shows the transfer characteristics of the P-HEMT. At low drain bias (such as *V*_DS_ = 1 V), the transfer curves of C-HEMT and P-HEMT are coincident well and the threshold voltage difference is less than 0.05 V. However, with the increasing of *V*_DS_, the *V*_TH_ of C-HEMT decreases obviously while *V*_TH_ of P-HEMT slightly reduced. Typically, the *V*_TH_ decrease from *V*_DS_ = 1 V to *V*_DS_ = 50 V is 0.59 V for C-HEMT and 0.1 V for P-HEMT, as shown in Figure 5b. The significantly decreased *V*_TH_ for C-HEMT will lead to false turn-on at high drain bias (typically, from off-state to on-state), which is not acceptable for practical application. However, from the results, it can be deduced that the P-HEMT with more stable *V*_TH_ can be contributed to alleviating this problem very well.

**Figure 6:**
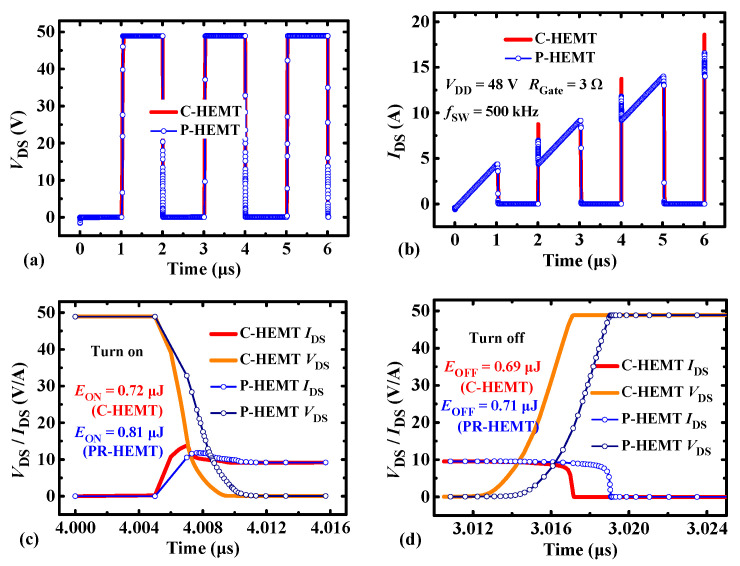
The switching transient comparison between C-HEMT and P-HEMT by double-pulse simulation. (**a**) *V*_DS_ voltage waveforms; (**b**) *I*_DS_ current waveforms; (**c**) turn on transient at ~10 A *I*_DS_ current; (**d**) turn off transient at ~10 A *I*_DS_ current.

**Figure 7:**
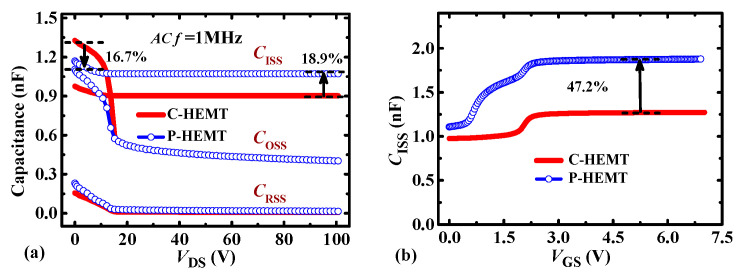
Comparison of (**a**) capacitance-*V*_DS_, and (**b**) *C*_ISS_-*V*_GS_ characteristics between C-HEMT and P-HEMT.

To illustrate the impact of the PR p-GaN layer on transient behavior, the simulation using a double pulse circuit is carried out, as shown in Figure 6. Compared with C-HEMT, the calculated turn-on loss and turn-off loss of P-HEMT are increased by 0.09 μJ and 0.02 μJ at 500 kHz respectively, and the total switching loss is increased by less than 7.8%. It can be inferred the increased switching loss mainly results from the increase of the input capacitance *C*_ISS_. As shown in Figure 7, it can be seen that the off-state and on-state input capacitance *C*_ISS_ is increased by 18.9% and 47.2%, respectively. In addition, as shown in Figure 7a, the *C*_OSS_ at high-drain bias (*V*_DS_ > 15 V) is the same as C-HEMT, and the output capacitance *C*_OSS_ at a low-drain bias (*V*_DS_ < 15 V) is decreased by 16.7%, which mainly results from the depletion of 2DEG channel under the PR layer as stated in section II. The decrease of *C*_OSS_ at *V*_DS_ < 15 V is contributed to reducing the increment of switching loss.

### 3.2. Design Considerations of P-HEMT

This section mainly discusses the impact of PR p-GaN layers’ thickness, length, and doping concentration on the BV and *V*_TH_ stability.

**Figure 8:**
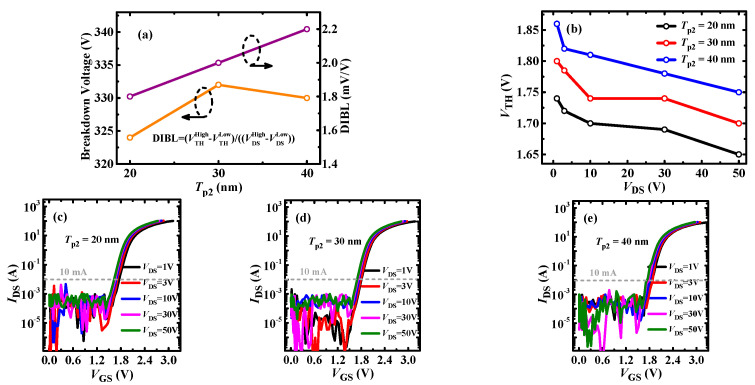
(**a**) Breakdown voltage (BV) and DIBL versus PR thickness; (**b**) the threshold voltage *V*_TH_ (at *I*_DS_ = 10 mA) depending on *V*_DS_; the transfer characteristics of P-HEMT with *T*_p2_ = (**c**) 20 nm; (**d**) 30 nm; (**e**) 40 nm.

**Figure 9:**
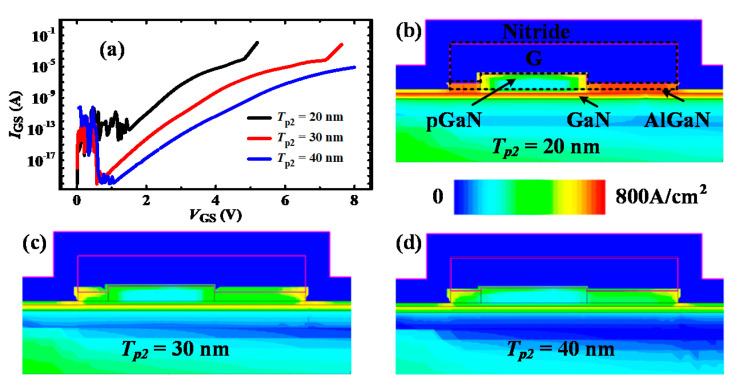
(**a**) The *I*_GS_-*V*_GS_ characteristics of P-HEMT with different *T*_p2_; the current distribution at the gate part for *T*_p2_ = (**b**) 20 nm, (**c**) 30 nm, (**d**) 40 nm.

Figure 8 shows the *V*_TH_ and BV results for different thicknesses of the PR p-GaN layer. As shown in Figure 8a, it can be seen that the DIBL value is increased with PR p-GaN layer thickness. The DIBL parameter is defined as ($V_{TH}^{High}$ − $V_{TH}^{Low}$)/($V_{DS}^{High}$ − $V_{DS}^{Low}$) to represent the *V*_TH_ stability, and the smaller value symbolizes the more stable *V*_TH_. As shown in Figure 8b, it can be seen that the *V*_TH_ for different thickness PR layers from *V*_DS_ = 1 V to *V*_DS_ = 50 V decreases, but the difference is all less than 0.1 V, which indicates the high stable *V*_TH_ for P-HEMT. The log-scale transfer characteristics are as shown in Figure 8c–e. For the same drain bias, the *V*_TH_ slightly increases (≤0.05 V) with the thickness of the PR p-GaN layer, which mainly results from the 2DEG depletion under the PR p-GaN layer. In addition, it can be observed that the BV is all larger than 320 V, which indicates the impact of the PR p-GaN layer’s thickness on BV is negligible. However, for a smaller thickness PR p-GaN layer, the gate-to-source breakdown voltage can be reduced. Figure 9a shows the *I*_GS_—*V*_GS_ characteristics for 20/30/40 nm PR p-GaN layer, and it can be seen that the *I*_GS_ for *T*_p2_ = 20 nm abruptly increases when *V*_GS_ is larger than 5.1 V. To explore the origin of the abrupt *I*_GS_, the current distribution of the three thickness PR p-GaN layer devices are plotted, as shown in Figure 9b–d. For the device with *T*_p2_ = 20 nm, the current density from the PR p-GaN layer to the 2DEG channel is larger than the normal thickness p-GaN layer. This indicates the high gate current mainly results from the breakdown of the PR p-GaN layer. As a comparison, the gate current density for *T*_p2_ = 30/40 nm is very small. Based on the above analysis, it can be deduced that the PR p-GaN layer thickness should be taken into careful consideration in the design to avoid gate breakdown.

**Figure 10:**
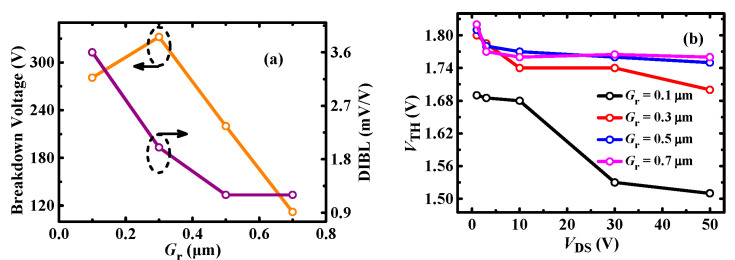
(**a**) BV and DIBL, (**b**) *V*_TH_ (at *I*_DS_ = 10 mA) depending on *V*_DS_ for different *G*_r_ length.

**Figure 11:**
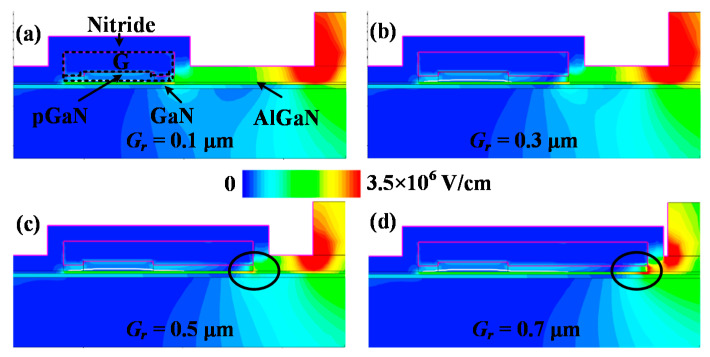
The electric field distribution of P-HEMT for Gr = (**a**) 0.1 μm; (**b**) 0.3 μm; (**c**) 0.5 μm; (**d**) 0.7 μm at breakdown voltage.

Figure 10 shows the impact of PR p-GaN layer length on the BV and *V*_TH_ characteristics. It can be seen that DIBL decreases with *G*_r_, which indicates the *V*_TH_ stability is increased. However, the DIBL tends to be stable and the BV decreases when *G*_r_ is larger than 0.5 μm. The decrease of the BV mainly results from the high electric field at the drain-side of the PR p-GaN layer, as shown in Figure 11. Therefore, it can be deduced that the PR p-GaN layer length should be in a reasonable range to get a good trade-off for *V*_TH_ stability and high BV. For the 100 V p-GaN HEMT discussed in this paper, the 0.3~0.5 μm PR p-GaN layer is recommended.

**Figure 12:**
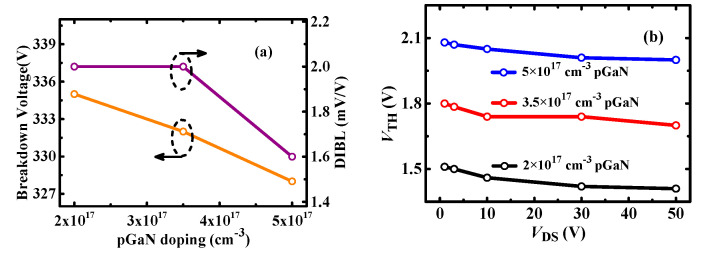
(**a**) BV and DIBL, (**b**) *V*_TH_ (at *I*_DS_ = 10 mA) depending on *V*_DS_ for different p-GaN doping density.

**Figure 13:**
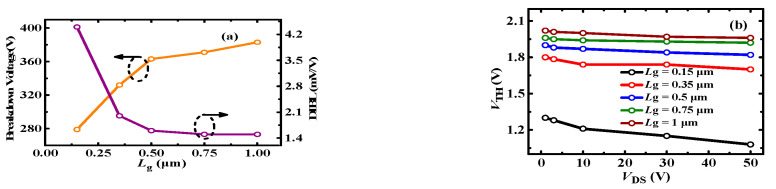
(**a**) BV and DIBL, (**b**) *V*_TH_ depending on *V*_DS_ for different gate lengths.

Figure 12 shows the impact of p-GaN doping density on the *V*_TH_ and BV. It can be observed that the p-GaN doping density mainly determines the magnitude of *V*_TH_, and it makes a negligible effect on BV and DIBL. Figure 13 shows the *V*_TH_ and BV characteristics of P-HEMT with different gate lengths *L*_g_. It can be seen that longer gate length induces higher *V*_TH_, lower DIBL, which means longer gate length is contributed to making *V*_TH_ more stable. In addition, longer gate length induces higher BV, which mainly results from the electric field modulation. However, longer gate length will induce higher on-resistance. Therefore, the gate length should be taken into careful consideration to get a better trade-off for *V*_TH_ stability, BV, and *R*_ON_.

## 4. Conclusions

This paper proposes a novel p-GaN HEMT with a PR p-GaN layer to improve BV and *V*_TH_ stability. The device features a PR p-GaN layer compared with conventional p-GaN HEMT. At high drain bias, the two-dimensional electron gas channel under the PR p-GaN layer is depleted to withstand *V*_DS_, thereby contributing to improving the BV and *V*_TH_ stability. Compared with the C-HEMT, simulation results show that the breakdown voltage is improved by 120%, and the *V*_TH_ stability changing with *V*_DS_ is increased by 490% (the decrease of *V*_TH_ at 50 V for P-HEMT and C-HEMT are 0.1 V and 0.59 V respectively). The static transfer and output characteristics are the same as the C-HEMT, and the total switching loss at 500 kHz is increased less than 7.8%. In addition, we investigated the impact of the PR layers’ length, thickness, doping density on the performance.

## Figures and Tables

**Table 1:** Device parameters specification.

Symbols	Definitions	Typical Value
*L*_S_	Source length	0.7 μm
*L*_G_	Gate length	0.35 μm
*L*_D_	Source-to-gate length	0.7 μm
*L*_GS_	Source-to-gate length	0.4 μm
*L*_DS-C_	C-HEMT Gate-to-drain length	1.95 μm
*L*_DS-PR_	P-HEMT Gate-to-drain length	1.95 μm
*L*_SFP-C_	C-HEMT Source-field-plate length	0.8 μm
*L*_SF-PR_	P-HEMT Source-field-plate length	(0.8—*G*_r_) μm
*L*_DFP_	Drain-field-plate length	0.25 μm
*t*_SiN_	Thickness of SiN	80 nm
*t*_SiO2_	Thickness of SiO_2_	270 nm
*G*_L_	The left PR p-GaN length	0.1 μm
*G*_R_	The right PR p-GaN length	0.3 μm
*T*_p2_	Thickness of PR p-GaN	30 nm
*T*_p1_	Thickness of p-GaN	50 nm
*t*_ba_	Thickness of barrier	12.5 nm
*t*_ch_	Thickness of channel	20 nm
*t*_bu_	Thickness of buffer	2 μm
*t*_nu_	Thickness of nucleation	10 nm
*t*_sub_	Thickness of substrate	550 μm
*t*_gate_	Thickness of Schottky gate	100 nm
*χ*_ba_	Al composition of barrier	25%
*χ*_bu_	Al composition of buffer	5%
*W*_G_	Work-function of the gate	4.8 eV
*N*_DT1_	Nitride/AlGaN trap density	3 × 10^13^ cm^−^^2^ (*E*_C_ − 0.4 eV) [24]
*N*_DTC_	Channel UID concentration	1 × 10^15^ cm^−^^3^
*N*_AT1_	Buffer acceptor trap density	3 × 10^16^ cm^−^^3^ (*E*_V_ + 0.9 eV) [17]
*N*_DT2_	Buffer donor trap density	1.3 × 10^15^ cm^−^^3^ (*E*_C_ − 0.11 eV)
*N*_AT2_	Silicon/AlN acceptor trap density	3 × 10^13^ cm^−^^2^ (*E*_C_ − 1.7 eV)
*N*_p-GaN_	Activated Mg Doping	3.5 × 10^17^ cm^−^^3^

## Data Availability

Not applicable.
